# Delayed recovery of consciousness from anesthesia due to exacerbation of hydrocephalus caused by a ventriculoperitoneal shunt malfunction during general anesthesia in the prone position: a case report

**DOI:** 10.1186/s40981-024-00736-x

**Published:** 2024-08-20

**Authors:** Yosuke Miyamoto, Takashi Kawasaki, Shingo Nakamura, Naoyuki Hirata

**Affiliations:** Department of Anesthesiology, Kumamoto University Hospital, 1-1-1, Honjo, Chuo-Ku, Kumamoto, 860-8556 Japan

**Keywords:** Delayed recovery from anesthesia, Ventriculoperitoneal shunts, Prone position

## Abstract

**Background:**

Dysfunction of ventriculoperitoneal (VP) shunts can lead to decreased levels of consciousness. We report a case of delayed emergence from anesthesia due to the malfunction of a VP shunt during neurosurgery in the prone position.

**Case presentation:**

A 75-year-old male with a history of VP shunt for a fourth ventricle obstruction underwent cerebral vascular anastomosis in the prone position. His preoperative level of consciousness was clear. The surgery under general anesthesia was completed without any particular issues. After discontinuation of anesthesia, the patient did not awaken for over an hour. Postoperative CT revealed exacerbated hydrocephalus, likely from VP shunt occlusion. After pumping the reservoir of the VP shunt, the patient regained consciousness. He was extubated and discharged from ICU on the second postoperative day with no neurological issues.

**Conclusion:**

For surgical patients with a VP shunt, anesthesia management must consider the risk of shunt malfunction due to patient positioning.

## Background

Hydrocephalus results from the distortion of normal cerebrospinal fluid (CSF) dynamics, leading to CSF accumulation and ventricular dilation [1]. Ventriculoperitoneal (VP) shunting, which reduces intracranial pressure by diverting CSF to the peritoneal cavity, is often used as the primary treatment for hydrocephalus [2]. VP shunts have been in use for many years, but despite significant advances in valve design and catheters, complications and failures occur relatively frequently [3]. VP shunts are prone to problems such as infection, migration, obstruction, and pseudocyst formation, leading to shunt failure and hydrocephalus [2–6].

When performing surgery on a patient with a VP shunt, the location of the shunt and the flow of CSF should be considered. There have been reports on the perioperative management of patients with VP shunts undergoing surgery [7, 8]. Conversely, there are few reports on intraoperative shunt failure, hydrocephalus, or other complications in surgical patients with VP shunts.

Here we report a case of shunt malfunction caused by prone positioning during neurosurgery, resulting in hydrocephalus and delayed awaking from anesthesia.

## Case presentation

**Fig. 1:**
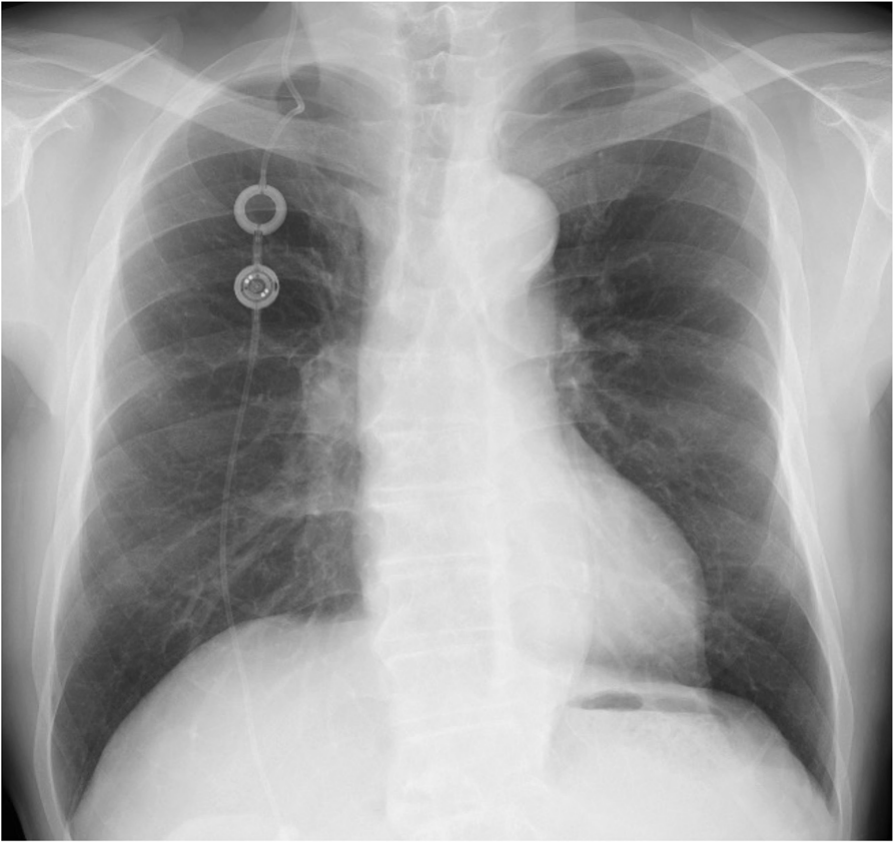
Preoperative chest X-ray. Ventriculoperitoneal shunt reservoir and valve located anteriorly in the thoracic region

The patient is a 75-year-old man (161 cm, 63.5 kg) who visited a nearby clinic due to the onset of dizziness, gait disturbance, and cognitive impairment. Head CT and MRI revealed a tumor in the fourth ventricle and non-communicating hydrocephalus. A VP shunt with a reservoir and valve (proGAV™ shunt system, BBraun, Germany) was implanted to treat the hydrocephalus (Fig. 1), resulting in improvements in gait disturbance and cognitive function. Upon thorough examination, the patient was diagnosed with a thrombosed aneurysm of the right posterior inferior cerebellar artery. Aneurysm trapping and cerebral revascularization surgery were scheduled. The patient, who is on oral medication for diabetes, had no other cardiovascular complications.

**Fig. 2:**
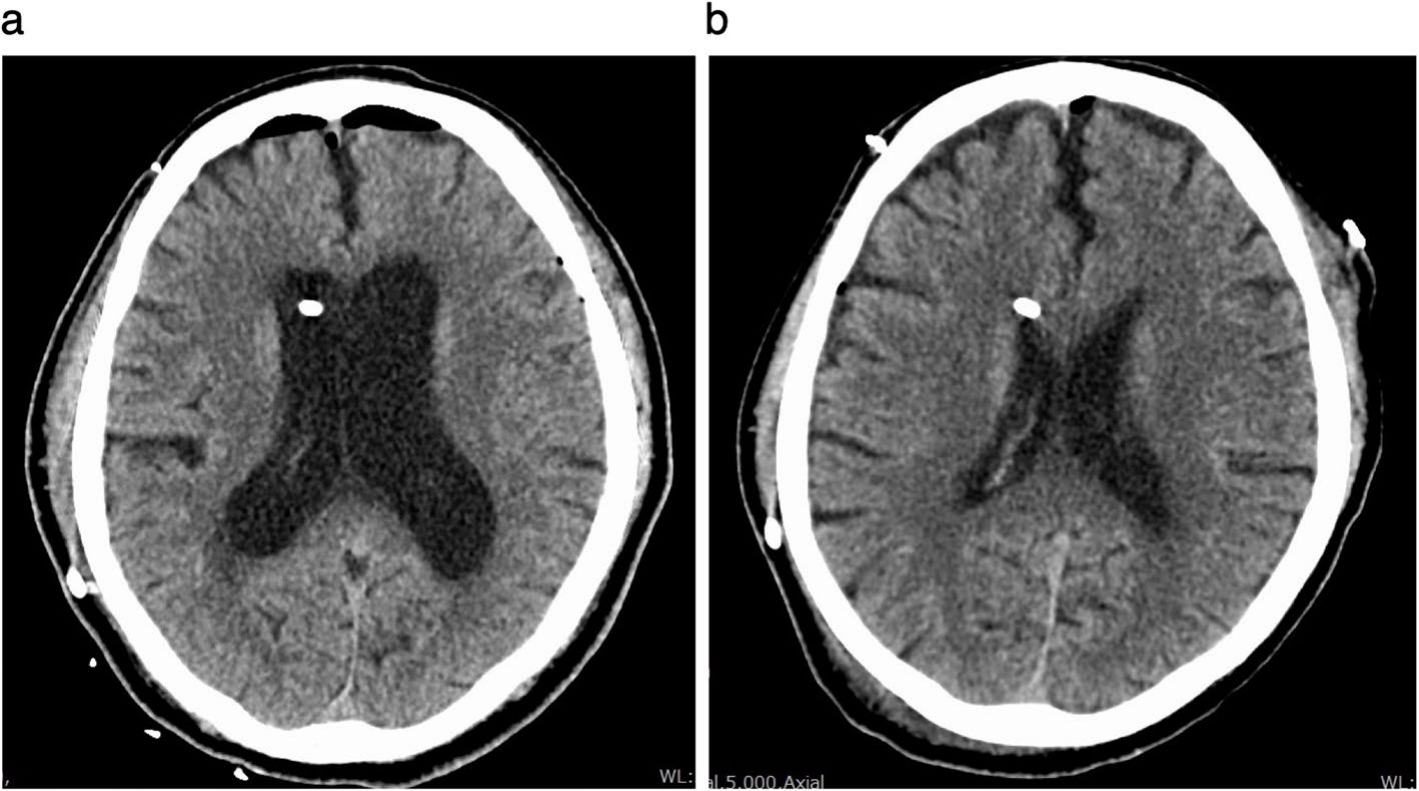
Head CT at just after surgery (**a**) and at the second postoperative day (**b**) after extubation

General anesthesia with total intravenous anesthesia was planned to monitor motor and somatosensory evoked potentials during surgery. Anesthesia induction was performed with propofol at an effect-site concentration of 3 µg/mL by target-controlled infusion, remifentanil at 0.2 µg/kg/min, fentanyl at 100 µg, and rocuronium at 40 mg. After tracheal intubation, the patient was placed in the prone position. Intraoperatively, TCI was used to adjust the propofol dosage and maintain a BIS value of 40–60. Respiratory and circulatory status remained stable during surgery. The operative time was 692 min, and the fluid balance was plus 2140 mL. After the surgery, spontaneous breathing resumed following the cessation of anesthetic agents, but the patient did not regain consciousness for over an hour. We did not detect any abnormal electroencephalogram findings on the BIS monitor. Blood gas analysis showed no significant abnormalities and body temperature was normal (37.0 °C). The possibility of a cerebrovascular event was considered. A head CT scan was performed with the patient remaining intubated. The CT scan revealed a worsening of hydrocephalus (Fig. 2a). It was hypothesized that the prone position had caused a VP shunt obstruction, exacerbating the hydrocephalus. Pumping the reservoir of the VP shunt resulted in the patient promptly opening his eyes and improving to a Glasgow Coma Scale of 9 (GCS, Eye, 4; Verbal, intubation; Motor, 4). The patient opened his eyes but was unable to follow commands and attempted to remove the tracheal tube himself. He was re-sedated with propofol and dexmedetomidine, with plans to awaken and extubate him after the hydrocephalus improved. The patient was admitted to the ICU, and the CSF outflow resistance on the VP shunt valve was changed from 14 to 10 cmH_2_O to increase CSF flow. The CT scan on the day after surgery showed insufficient improvement in hydrocephalus, so sedation was continued. During sedation management in the ICU, the hemodynamics were stable, and the respiratory condition was managed with pressure support ventilation (support pressure, 5 cmH_2_O; PEEP, 5 cmH_2_O; FiO_2_, 0.3), with a PaO_2_/FiO_2_ ratio of approximately 400 mmHg. The CT scan on the second postoperative day confirmed improvement in hydrocephalus (Fig. 2b), allowing us to stop sedation. After awakening, the patient could follow commands (GCS of 11, Eye,4; Verbal, intubation; Motor, 6), and no other neurological abnormalities were confirmed, so the patient was extubated and discharged from the ICU to the ward.

## Discussion

We reported a case where prolonged prone positioning led to VP shunt obstruction, worsening hydrocephalus, and delayed emergence from anesthesia. There have been reports of increased intra-abdominal pressure during laparoscopic surgery causing VP shunt malfunction [9]. However, to our knowledge, no reports have identified intraoperative positioning as a factor in VP shunt obstruction.

Several factors can induce delayed emergence from anesthesia [10, 11]. Residual effects of sedatives are unlikely to be the cause in this case because of intraoperative BIS values between 40 and 60, normal liver and renal function, and normal preoperative hypoalbuminemia or hypothermia. Blood gas data, glucose, and electrolytes were also within normal ranges. Based on differential diagnosis, we considered that a complication in the central nervous system might have occurred. Postoperative CT revealed hydrocephalus despite the presence of a VP shunt. To promote the flow of CSF, the unidirectional reservoir of the shunt was pumped. A few minutes after pumping the reservoir, the patient’s eyes opened. This suggests that the VP shunt might not have functioned intraoperatively, leading to hydrocephalus and delayed recovery of consciousness after the cessation of anesthetics.

The detailed mechanisms of VP shunt occlusion remain unclear. The VP shunt may have kinked at the cervical level due to the forward flexion of the head during occipital surgery. Another possibility is that the prone position itself may have occluded the VP shunt tube. The VP shunt tube was placed subcutaneously in the anterior thoracic region (Fig. 1) and may have been compressed by the body. Other possibilities include increased intra-abdominal pressure due to the prone position, which may have obstructed the CSF flow.

The preoperative CSF outflow resistance was set at 14 cmH_2_O. This value may have been appropriate because the patient’s preoperative level of consciousness was normal [12]. Therefore, it is unlikely that the CSF outflow resistance contributed to the delayed recovery of consciousness after anesthesia.

While we had recognized that the patient had a VP shunt, the possibility of intraoperative obstruction or malfunction of the VP shunt due to the surgical position had not been considered. Previously, increased intra-abdominal pressure during laparoscopic surgery has been shown to induce malfunction of a VP shunt [9]. To avoid this complication, several reports have demonstrated the usefulness of transcranial Doppler for CSF flow monitoring [7, 8]. In the perioperative management of patients with VP shunts, it is important to consider the risk of shunt malfunction perioperatively, protect the shunt route, and monitor CSF flow with transcranial Doppler intraoperatively, depending on the situation.

In conclusion, we reported a case of VP shunt malfunction and exacerbation of hydrocephalus during neurosurgery under prolonged prone positioning, which resulted in a delayed recovery of consciousness after anesthesia. It is important to manage anesthesia with the possibility of intraoperative shunt malfunctions in mind, depending on the position of the patient with a VP shunt implantation.

## Data Availability

Data sharing is not applicable to this article as no datasets were generated or analyzed during the current study.

## Abbreviations

VP: Ventriculoperitoneal
CT: Computed tomography
CSF: Cerebrospinal fluid
TCI: Target-controlled infusion
BIS: Bispectral index
